# Omega-3 Fatty Acid Deficiency during Brain Maturation Reduces Neuronal and Behavioral Plasticity in Adulthood

**DOI:** 10.1371/journal.pone.0028451

**Published:** 2011-12-07

**Authors:** Harsharan Singh Bhatia, Rahul Agrawal, Sandeep Sharma, Yi-Xin Huo, Zhe Ying, Fernando Gomez-Pinilla

**Affiliations:** 1 Department of Integrative Biology and Physiology, University of California Los Angeles, Los Angeles, California, United States of America; 2 Department of Neurosurgery, University of California Los Angeles, Los Angeles, California, United States of America; 3 UCLA Brain Injury Research Center, University of California Los Angeles, Los Angeles, California, United States of America; 4 Department of Chemical and Biomolecular Engineering, University of California Los Angeles, Los Angeles, California, United States of America; Rikagaku Kenkyūsho Brain Science Institute, Japan

## Abstract

Omega-3-fatty acid DHA is a structural component of brain plasma membranes, thereby crucial for neuronal signaling; however, the brain is inefficient at synthesizing DHA. We have asked how levels of dietary n-3 fatty acids during brain growth would affect brain function and plasticity during adult life. Pregnant rats and their male offspring were fed an n-3 adequate diet or n-3 deficient diets for 15 weeks. Results showed that the n-3 deficiency increased parameters of anxiety-like behavior using open field and elevated plus maze tests in the male offspring. Behavioral changes were accompanied by a level reduction in the anxiolytic-related neuropeptide Y-1 receptor, and an increase in the anxiogenic-related glucocorticoid receptor in the cognitive related frontal cortex, hypothalamus and hippocampus. The n-3 deficiency reduced brain levels of docosahexaenoic acid (DHA) and increased the ratio n-6/n-3 assessed by gas chromatography. The n-3 deficiency reduced the levels of BDNF and signaling through the BDNF receptor TrkB, in proportion to brain DHA levels, and reduced the activation of the BDNF-related signaling molecule CREB in selected brain regions. The n-3 deficiency also disrupted the insulin signaling pathways as evidenced by changes in insulin receptor (IR) and insulin receptor substrate (IRS). DHA deficiency during brain maturation reduces plasticity and compromises brain function in adulthood. Adequate levels of dietary DHA seem crucial for building long-term neuronal resilience for optimal brain performance and aiding in the battle against neurological disorders.

## Introduction

The causes of most neurological disorders are indefinite and characterized by multiple components, in which the interaction of the environment with the genome likely plays a major role. Dietary factors are garnering special recognition as important modifiers of brain function and plasticity, and mental heath [1]. Anxiety and depression disorders have no clear causes and are commonly encountered in industrialized societies, posing high health care and economic burdens. In addition to affecting individuals at all stages of life, an alarmingly increasing number of young adults suffer from anxiety disorders [2] making it imperative to develop therapeutic strategies to moderate the incidence of mood disorders. New studies emphasize the quality of the diet as an important factor affecting the occurrence of mood disorders in a large population of adults [3]; however, poor knowledge on the molecular mechanisms involved has delayed the implementation of diet as a strategy for the prevention or treatment of mood disorders.

The capacity of the n-3-fatty acid docosahexaenoic acid (DHA; 22∶6n-3) to promote brain plasticity and cognitive function is starting to be recognized [4]. DHA is an important component of neural membranes that accumulates rapidly in tissue during infancy [5]. Neither brain nor body can synthesize DHA by itself and it must be obtained from diet [6]. During prenatal development, DHA is delivered to the brain by maternal source via uteroplacental circulation [7], emphasizing the importance of maternal supply of n-3 fatty acids for the neurological function of the offspring. In turn, n-6 fatty acids generally compete with n-3 fatty acids for membrane occupancy such that the reciprocal relationship between n-3 and n-6 fatty acids is altered in DHA deficient conditions, imposing a risk for neuronal function [8], [9].

Brain-derived neurotrophic factor (BDNF) is important for brain function and plasticity throughout lifespan. It exerts its biological function through binding to its receptor TrkB, which initiates multiple signaling cascades [10]. Dysfunction in BDNF signaling may be implicated in the pathophysiology of anxiety and depression [11], [12], [13]. The action of BDNF has been linked to molecules important for the control of brain energy metabolism and anxiety-like behaviors such as neuropeptide Y (NPY) and glucocorticoid receptor (GR) [14].

Dietary factors are intrinsically related to metabolism, and new evidence emphasizes the potential link between brain metabolism and synaptic plasticity [15]. These interactions can be particularly prevalent during brain development, modulating the risk for psychiatric disorders in adulthood [16], [17]. Therefore, it is important to understand how metabolic elements such as the insulin system can affect the substrates of mental function. The current study suggests the interesting possibility of diet as a modulator of brain metabolism and behavioral plasticity and may shed light on a new, non-invasive, and cost-effective therapeutic solution for mental illnesses.

## Results

### Effects of n-3 fatty acid dietary manipulation on Anxiety-like behavior

**Figure 1:**
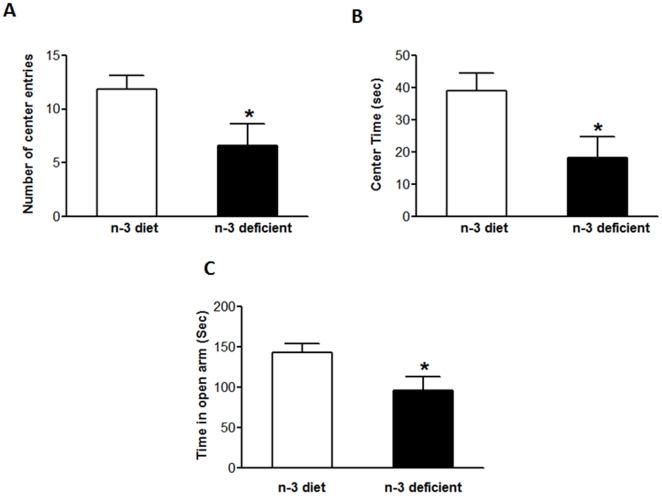
Effect of dietary n-3 fatty acid deficiency on anxiety-like behavior. Open field showed a decrease in number of center entries (p<0.05) (A) and center time (p<0.05) (B) in n-3 deficient group compared to n-3 diet group. (C) EPM showed a decrease in time spent in open arm in n-3 deficient as compared to n-3 diet. Values are expressed in mean ±SEM. *p<0.05 Vs n-3 diet.

The open field (OF) and elevated plus maze (EPM) studies were performed to assess anxiety-like behavior. Omega-3 def rats showed a significantly decreased number of center entries as compared to n-3 diet group (t_10_ = 2.222, *p* = 0.0433) as shown in Fig. 1A. The time spent at the center of the arena was significantly decreased in n-3 def rats as compared to n-3 diet rats as shown in Fig. 1B (t_10_ = 2.398, *p* = 0.0310). To further confirm the anxiogenic effects of n-3 def diet we performed the elevated plus maze test. In fig. 1C, the n-3 def group significantly decreased the time spent in open arms as compared to the n-3 diet group (t_10_ = 2.346, *p* = 0.0342).

### Effects of n-3 deficiency on the levels of fatty acids in brain

**Figure 2:**
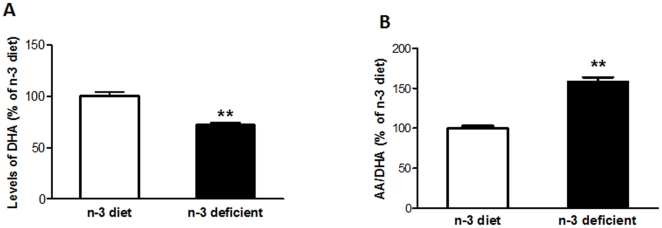
Effect of dietary n-3 fatty acid deficiency on DHA and AA levels in brain. (A) A significant decrease (p<0.01) in the levels of DHA in brain in n-3 deficient fed animals as compared to n-3 diet animals. (B) An increase (p<0.01) in the ratio of arachidonic acid (AA) to docosahexenoic acid (DHA) in n-3 deficient animal group as compared to n-3 diet. Values are expressed in mean ±SEM. **p<0.01 Vs n-3 diet.

To assess the effects of n-3 deficient diet, we measured the levels of various fatty acids in brain by using gas chromatography as shown in Table 1. We found a significant decrease in the levels of DHA in the animal group fed on n-3 deficient diet as compared to the n-3 fed diet counterpart (t_10_  =  4.884, *p* =  0.0006) (Table 1. C22∶6n-3; Fig. 2A). The ratio of the n-6 fatty acid arachidonic acid (AA) versus n-3 fatty acid DHA (AA/DHA) was significantly increased in the n-3 deficient animals as compared to n-3 diet animals (t_10_ = 10.33, *p*<0.0001) (Table 1. C18∶2n-6/C22∶6n-3, Fig. 2B).

**Table 1:** Fatty acid composition in brain tissue.

Fatty acid	n-3 diet	n-3 deficient
C14:0	0.24±0.03	0.27±0.021
C16:0	18.61±0.73	20.26±1.03
C16:1	0.52±0.10	0.93±0.44
C18:0	19.59±0.65	20.34±0.95
C18:1	14.86±0.52	14.46±0.58
C18:2n-6	0.64±0.10	0.65±0.06
C20:0	0.57±0.14	0.49±0.01
C20:1	1.36±0.14	1.83±0.69
C20:2	0.51±0.13	0.34±0.05
C20:3n-6	0.75±0.10	0.51±0.09
C20:4n-6	8.68±0.29	9.96±0.59
C22:4n-6	5.70±2.41	4.67±1.53
C24:0	0.82±0.10	1.12±0.06
C22:6n-3	14.81±0.62	10.70±0.46a
C18:2n-6/C22:6n-3	0.58±0.01	0.93±0.03a

Each parameter is presented as the percentage mean relative to total fatty acids (± SEM). Statistically significant changes are represented ^a^p<0.01 compared with n-3 diet. Data were analyzed by using two-tailed unpaired t-test.

### Effects of n-3 deficiency on molecules associated with anxiety-like behavior

**Figure 3:**
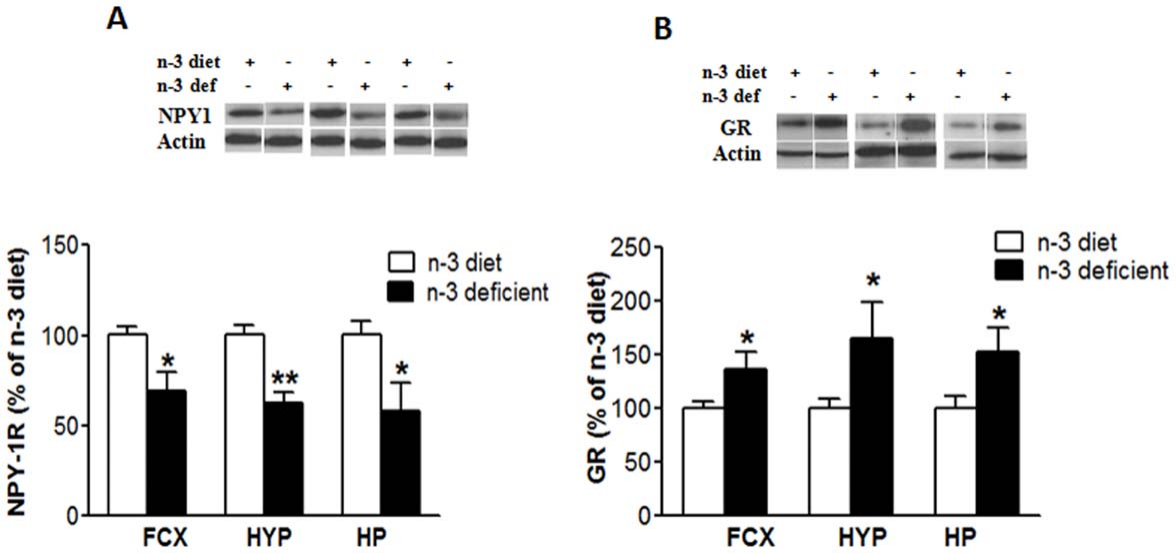
Effects of n-3-fatty acid dietary deficiency on proteins related to anxiety-like behavior. The n-3 deficient diet significantly decreased protein levels of Neuropeptide Y (NPY) 1 receptor (A), and increased glucocorticoid receptor (GR) (B), in frontal cortex, hypothalamus, and hippocampus. Values are expressed in mean ±SEM. *p<0.05, **p<0.01Vs n-3 diet.

The anxiety-reducing effects of NPY and the anxiety-enhancing effects of antagonists of NPY receptors are fairly well-documented, providing strong evidence for NPY's role in modulating anxiety responses. We checked the modulating effects of n-3 deficient diet and n-3 diet on the levels of NPY1 receptor in specific brain regions. We found a significant decrease in frontal cortex (t_10_ = 2.957, *p* = 0.0144), hypothalamus (t_10_ = 4.362, *p* = 0.0014) and hippocampus (t_10_ = 2.608, *p* = 0.0261), when rats were fed an n-3 deficient diet (Fig. 3A). We also wanted to determine whether early dietary events induced persistent modifications in the levels of glucocorticoid receptor (GR), a molecule that responds to stress related situations. We found that the n-3 deficient group showed increased percentage levels of GR in the frontal cortex (t_10_ = 2.415, *p* = 0.0364), hypothalamus (t_10_ = 2.494, *p* = 0.0317) and hippocampus (t_10_ = 2.326, *p* = 0.0424), as compared to the n-3 diet counterpart (Fig. 3B).

### Effects of n-3-deficiency on molecules associated with synaptic plasticity

**Figure 4:**
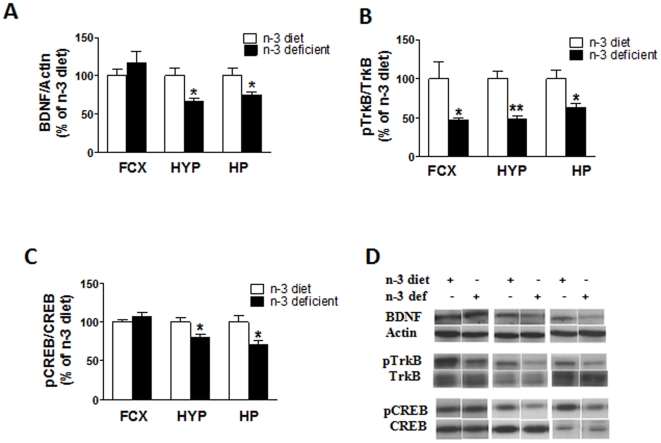
Effects of n-3-fatty acid dietary deficiency on proteins related to synaptic plasticity. (A) BDNF levels were significantly decreased in the hypothalamus and the hippocampus in n-3 deficient animals compare to n-3 diet animals (p<0.05). (B) Phospho-TrkB (pTrkB) levels showed significant decrease in frontal cortex (p<0.05), hypothalamus (p<0.01) and hippocampus (p<0.05) in n-3 deficient group compare to n-3 diet group. (C) pCREB levels were decreased in hypothalamus (p<0.05) and hippocampus (p<0.05) but not in front cortex in n-3 deficient rats compare to n-3 diet rats. (D) Representative western blot bands are shown for BDNF, pTrkB, TrkB, pCREB, CREB and actin in three different brain regions. Values are expressed in mean ±SEM. *p<0.05, **p<0.01 Vs n-3 diet.

In rats fed n-3 deficient diets, results showed a significant decrease on the levels of BDNF in the hypothalamus (t_10_ = 2.758, *p* = 0.0202) and hippocampus (t_10_ = 2.273, *p* = 0.0463) when compared to n-3 diet. In the frontal cortex (t_10_ = 0.6751, *p* = 0.5149), we observed no significant change between diets (Fig. 4A).

Previous studies have shown that young mice lacking functional full-length TrkB exhibited a markedly enhanced anxiety-like behavior as evidenced by their decreased explorative activity in the open field and elevated plus maze tests [20]. In our study, we observed a significant decrease in frontal cortex (t_10_ = 3.002, *p* = 0.0133), hypothalamus (t_10_ = 4.377, *p* = 0.0014) and hippocampus (t_10_ = 2.946, *p* = 0.0146) on the levels of phosphorylated TrkB in n-3 deficient group in all three regions respectively (Fig. 4B).

BDNF/TrkB-mediated signaling involve the MAP kinase and PI-3 kinase pathways leading to activation of cyclic AMP response element binding protein (CREB), which has been reported to be a key mediator of cell survival via initiation of transcription [21]. We assessed levels of phospo-CREB (pCREB) to further elucidate the effects of omega-3-fatty acid deficiency on BDNF signaling. We found that levels of pCREB significantly decreased in the hypothalamus (t_10_ = 2.406, *p* = 0.0369) and in the hippocampus (t_10_ = 2.563, *p* = 0.0282), while no significant changes were observed in the frontal cortex (t_10_ = 2.022, *p* = 0.0707) of rats fed an n-3 deficient diet as compared to n-3 diet rats (Fig. 4C).

### Association between levels of DHA and pTrkB receptor

**Figure 5:**
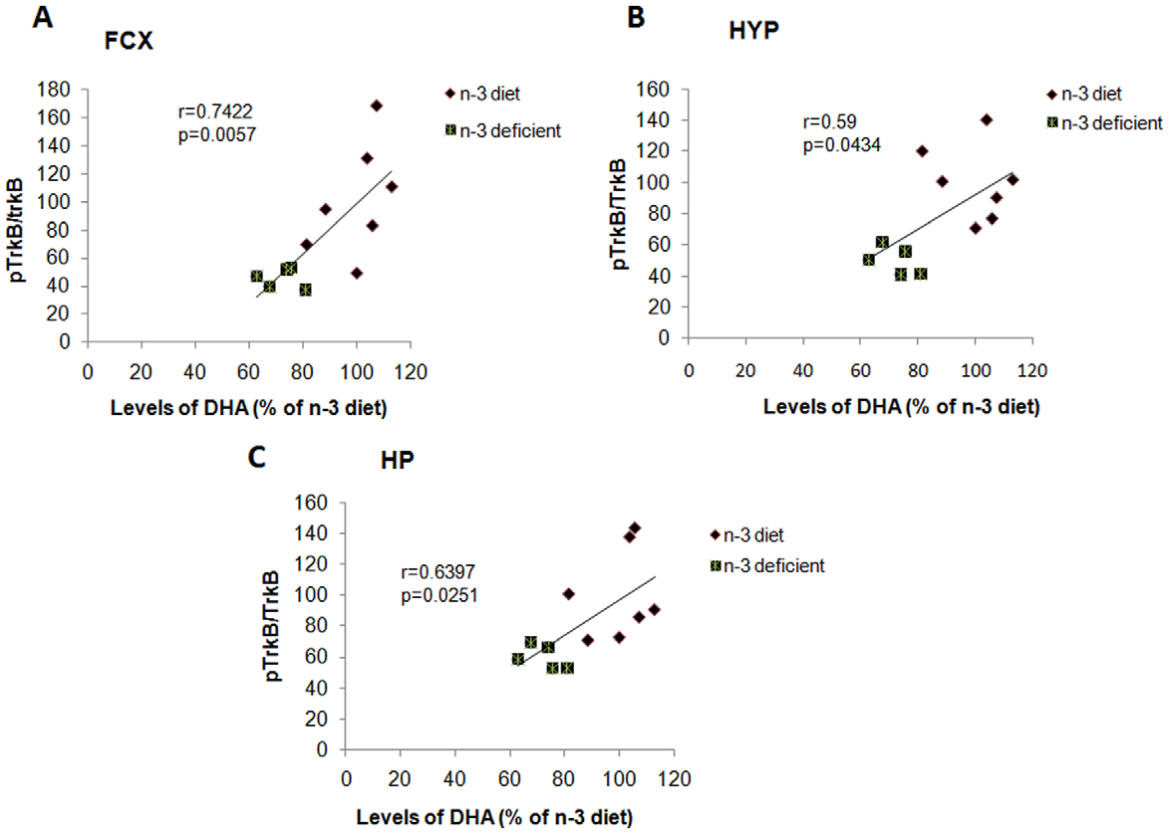
A significant positive correlation was observed between brain DHA levels and levels of pTrkB receptor in (A) frontal cortex (r = 0.74, p<0.01), (B) hypothalamus (r = 0.59, p<0.05), and (C) hippocampus (r = 0.63, p<0.05).

Given the importance of dietary n-3 fatty acid on BDNF signaling, we have correlated the levels of DHA to the levels of activated TrkB in the three brain regions. We found a positive correlation between the levels of DHA to the activated TrkB in frontal cortex (r = 0.7422, *p* = 0.0057; Fig 5A), hypothalamus (r = 0.59, *p* = 0.0434; Fig. 5B) as well as hippocampus (r = 0.6397, *p* = 0.0251; Fig. 5C).

### Molecules related to metabolic dysfunction

**Figure 6:**
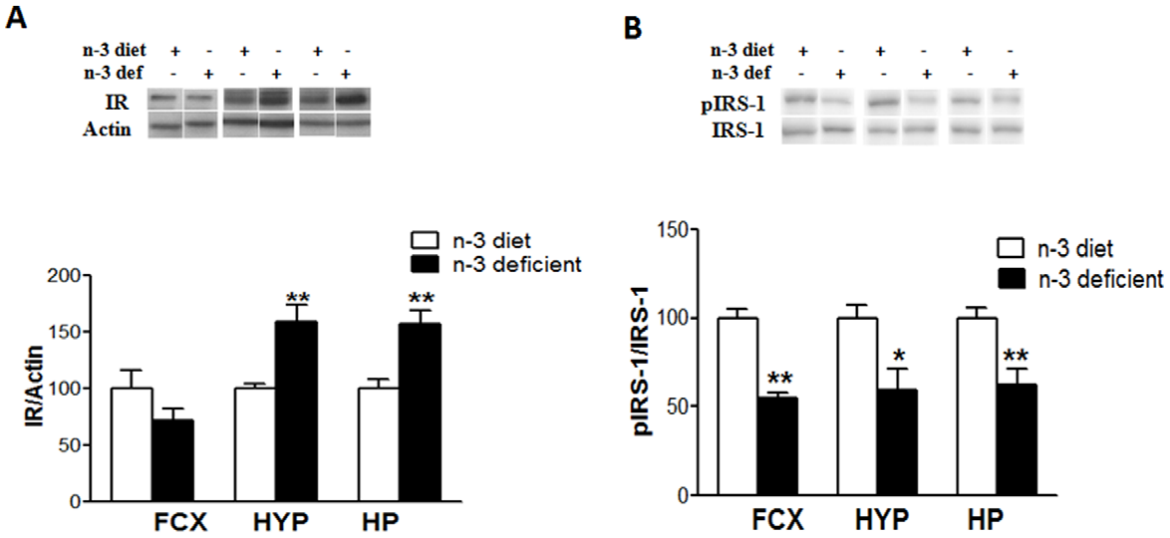
Effects of n-3-fatty acid dietary deficiency on protein levels of (A) insulin receptor, the hypothalamus and in hippocampus showed significant increases as compared to n-3 diet animals (p<0.01). (B) Phospho-insulin substrate receptor-1 (pIRS-1) showed significant decrease in frontal cortex (p<0.01), hypothalamus (p<0.05) and hippocampus (p<0.01) as compared to n-3 diet animals. Values are expressed in mean ±SEM. *p<0.05, **p<0.01 Vs n-3 diet.

Additionally, we have assessed a possible link between metabolic regulators and mood disorders. We selected the molecules insulin receptor (IR) and insulin receptor substrate-1 (IRS-1) for their potential role in metabolic disorders affecting mental function. We showed that an n-3 deficient diet differentially regulates the levels of insulin receptor in the three studied brain regions. A significant increase was observed in hypothalamus (t_10_ = 4.806, *p* = 0.0007) and and hippocampus (t_10_ = 4.068, *p* = 0.0023); on the contrary, a decreasing trend was observed in the frontal cortex (t_10_ = 1.3, *p* = 0.2229) (Fig. 6A). We found a significant decrease in the IRS-1 phosphorylation in the frontal cortex (t_10_ = 7.375, *p* <0.001), hypothalamus (t_10_ = 3.024, *p* = 0.0128) and hippocampus (t_10_ = 3.488, *p* = 0.0058) in the activation of IRS-1 (Fig. 6B).

## Discussion

The purpose of the present study is to understand how dietary n-3 fatty acids consumed during early development can influence the brain's capacity to endure challenges during adulthood. A rapid accretion of the polyunsaturated fatty acid DHA has been reported to occur during prenatal development in humans and rats [22]. In turn, our results indicate that the consumption of a diet deficient in DHA during gestation, lactation, and infancy increases the risk for anxiety-like behavior during adulthood, and suggest potential molecular mechanisms involved. BDNF signaling through its receptor TrkB changed proportionally to levels of brain DHA, such that animals with the lowest DHA showed the least index in TrkB signaling and an increase in the n-6/n-3 ratio. Given the importance of n-3 fatty in membrane fluidity, it is likely that lowered levels of DHA may have contributed to the reduction of TrkB signaling. In addition, the DHA deficient diet reduced markers of BDNF-related synaptic plasticity in the frontal cortex, hippocampus and hypothalamus. The DHA deficiency also resulted in altered levels of insulin receptor, which are important for maintaining metabolic homeostasis and cognition. These data emphasize the importance of DHA consumption during early development, which can determine the potential for plasticity and mental health during adulthood.

### Molecular mechanisms: DHA levels correlate with BDNF receptor signaling

A deficit of n-3 fatty acid significantly reduced the levels of BDNF in the hypothalamus and hippocampus of adult rats. Disruption in BDNF function has been implicated in the pathophysiology of psychiatric disorders such as depression [23], [24], [25], and most treatments against anxiety are associated with the action of BDNF and its receptor [26]. In our results, the level of activated TrkB positively correlated to brain DHA contents for all three studied brain regions, and the n-3 deficient diet reduced the activation of TrkB receptor. These results hold well with previous findings that mice lacking functional TrkB signaling, specifically in newborn neuronal populations, exhibit a markedly enhanced anxiety-like behavior as adults [27]. Furthermore, a recent study showed that an 11 base pair deletion in the *TrkB* promoter could promote anxiety related traits in human [28]. Furthermore, given that DHA is a structural component of the plasma membrane, reductions in DHA can have a direct influence in the function of the membrane. Disruptions in membrane fluidity can lower performance of transmembrane receptors, such as reducing signaling through TrkB. The increase in the ratio n-6/n-3 observed in our results might be indicative for a replacement of DHA by the n-6 arachidonic acid in the membrane, and our results showed that this ratio increase was associated with a reduction in TrkB signaling. As discussed below, DHA deficiency also affected levels of the insulin receptors and related signaling IRS-1, which may also relate to changes in the membrane fluidity.

In our studies, there was a reduction in the activation of CREB with the n-3 deficient diet in the hypothalamus and hippocampus. It has been shown that decreases in CREB phosphorylation and NPY expression in the central amygdala might be associated with anxiety-like behaviors in models of ethanol withdrawal in rats [29]. BDNF binding to its TrkB receptor leads to phosphorylation of TrkB and downstream proteins such as the transcription factor cyclic AMP-dependent response element binding protein (CREB). In turn, CREB regulates the expression of many genes, including BDNF [30], [31] and NPY-1 [32].

There were no significant changes in phosphorylation of CREB and BDNF levels in the frontal cortex, and these results differ from a previous study's results [33]. This apparent discrepancy might be due to the varying rat strain, methods used to measure the levels of BDNF, or regional differences. Accordingly, there could be differential vulnerability of different brain regions to the n-3 deficiency at the level of transcription factor CREB activation. CREB function is associated with the expression of BDNF as CREB phosphorylation is required to transcribe CREB-regulated genes including BDNF [31]. Despite the lack of net BDNF changes, there was a significant reduction in activated TrkB levels in the frontal cortex of rats fed the n-3 fatty acid deficient diet, suggesting that the n-3 deficiency during brain development could downregulate TrkB activity through BDNF-independent events. A recent study has shown that addition of zinc to cortical neurons cultured from mice carrying a null mutation of BDNF resulted in increased activation of TrkB similar to that of wild type controls, suggesting the activation of TrkB in the absence of BDNF [34].

### Anxiety-like behavior and metabolism

We assessed levels of NPY-1R based on its action providing resilience against anxiety and depression-like behavior [35], particularly throughout the frontal cortex and limbic regions [36]. Therefore, it is significant that early n-3 dietary manipulation affected NPY-R in the frontal cortex, hypothalamus and hippocampus in conjunction with the increase in anxiety-like behavior. The human orbitofrontal cortex receives reciprocal connections from the hippocampus, nucleus accumbens, and hypothalamus [37] and is thought to play a significant role in hedonic and emotional processes implicated in psychiatric disorders. The frontal cortex, together with hippocampus, amygdala and hypothalamus, are limbic regions forming part of well-defined anxiety and fear-related circuits in the forebrain [38]. Given that anxiety plays an important role in altering stress related behaviors; we evaluated the protein levels of glucocorticoid receptor as a marker for enhanced stress. We found increased levels of the GR reported in the three brain regions when animals were on n-3 deficient diet. Taken together, the decrease in NPY-1R and increase in GR in n-3 deficient rats may suggest that early DHA deficiency can hinder the ability to cope with challenges in adulthood, leading to anxiety-like behavior. An increasing line of evidence indicates that anxiety and stress are coupled with metabolic disturbances [1]. For example, in addition to anxiety and stress, NPY and GR have been involved in regulation of energy balance and appetite. Therefore, changes in NPY-1R and GR by the DHA deficient diet may also have implications for mechanisms that control food intake.

### A potential metabolic pathway to cognition

We found that n-3 dietary deficiency exerted differential effects on molecular systems vital for the regulation of metabolic disorders that compromise cognitive abilities. For example, a DHA deficient diet influenced the insulin signaling system as evidenced by increases in insulin receptor (IR) levels in the hippocampus and hypothalamus, and by reductions in phosphorylation of IR substrate (IRS)-1 in all three regions. Insulin resistance is a concept generally used to describe reduced insulin signaling in spite of high levels of insulin in the body. Our results seem to suggest that the lack of dietary n-3-fatty acids may predispose the brain to insulin resistance and may be considered a risk factor for diabetes. These studies emphasize how the maternal diet can program offspring growth and metabolic pathways, which can further alter lifelong susceptibility to metabolic disorders.

### Conclusions

**Figure 7:**
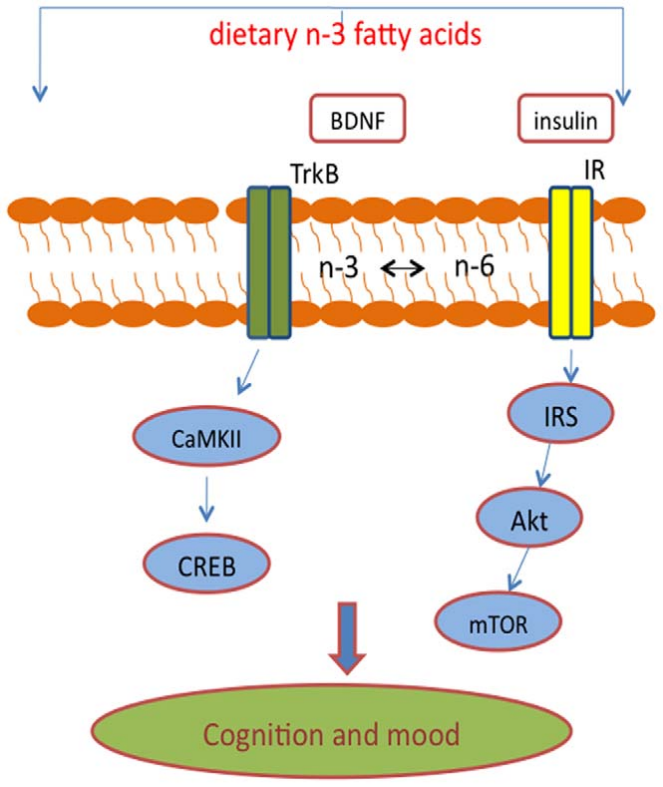
Schematic representation for potential pathways by which n-3 dietary deficiency may enhance vulnerability to cognitive and mood disorders. Reductions in plasma membrane DHA may disrupt signaling of membrane embedded receptors, such as the BDNF receptor TrkB (Fig. 4B) and the insulin receptor (IR; Fig. 6A). Dysfunction in TrkB signaling may influence downstream BDNF cascades such as CaMKII and CREB (Fig. 4C), leading to further dysfunction in the BDNF system, and ultimately increasing vulnerability for anxiety-like behavior. Given the interaction between foods, BDNF, synaptic plasticity, and metabolic pathways, the n-3 dietary deficiency may disrupt events related to the insulin receptor (IR) signaling pathways elements such as the IR substrate IRS-1, Akt and mTOR (Fig. 6B), which in turn, can affect BDNF-related synaptic plasticity leading to increased anxiety-behavior. Our results show that metabolic and behavioral pathways are both impacted by DHA deficiency.

The results are significant to understand how dietary n-3 fatty acids during brain growth can influence the brain's capacity to sustain challenges during adult life. Manipulations of the early environment can affect the expression of neurotrophins both during development and adulthood [39], [40], [41]. In our study, the contents of the DHA in the diet influenced markers of synaptic plasticity and energy metabolism in the frontal cortex, hippocampus, and hypothalamus. More dramatically, DHA contents were associated with levels of the BDNF signaling receptor TrkB in the different brain regions. Our results seem to illustrate a potential mechanism by which the consumption of DHA in infancy can help counteract the risk for anxiety-like behavior during adulthood (Fig. 7). The concept of “cognitive reserve” is generally used to describe the brain's capacity to build resistance to cope with future challenges; however, its bases are poorly understood. The results showing that the absence of an adequate diet during brain formation has profound consequences for adult brain plasticity are instrumental to better understand the neural basis for building cognitive reserve.

## Materials and Methods

### Experimental designs and tissue preparation

Female Sprague–Dawley rats were obtained on the 2nd day of pregnancy from Charles River (Portage, MI) weighing between 280 and 300 g. Animals were housed in cages and maintained in environmentally controlled rooms (22–24°C) with a 12-h light/dark cycle. They were divided into two dietary groups on a pseudorandom basis with the constraint that the two groups had the same mean body weight. One group of pregnant females was fed an n-3 fatty acid adequate diet and a second group was fed an n-3 fatty acid deficient diet, which are abbreviated as n-3 diet and n-3 def respectively throughout the study. Rats were maintained on these diets through gestation and lactation, and their male offspring were weaned to the same diet as their dams and maintained for 15 weeks. The two custom diets used were based on the composition of the American Institute of Nutrition diet and prepared commercially (Dyets, Bethlehem, PA) as previously described [18]. Both diets had the same basal macronutrients, vitamins, minerals, and basal fats (hydrogenated coconut and safflower oils). The only difference between the n-3 diet and n-3 def was the amount of n-3 fatty acids, which was achieved by adding 0.5% of flaxseed oil and 1.2% of docosahexaenoic acid (Nordic Naturals, Inc. Watsonville, CA, USA) to the n-3 diet to supply n-3 fatty acids (Table S1).

At the end of 15 weeks, the male offspring rats were subjected to open field and elevated plus maze tests to assess the anxiety-like behavior. A day after the behavioral tests, animals were killed by decapitation and the fresh tissues, including the frontal cortex, hypothalamus and hippocampus, were dissected, frozen in dry ice and stored at −70°C until use for biochemical analyses. Experiments were performed in accordance with the United States National Institutes of Health Guide for the Care and Use of Laboratory Animals, and were approved by the University of California at Los Angeles Chancellor's Animal Research Committee (ID: ARC 2001-164). The suffering and number of animals used were minimized.

### Behavioral Analyses

#### Open Field

The open field consisted of a 1.2 m diameter circular tank with 60 cm walls. An inner circle, 80 cm in diameter, was marked on the tank floor to serve as a central arena. Testing began when each rat was placed in the middle of the central arena and allowed to explore the field for 10 min. Rat behavior was recorded by an overhead camera. Measurement included time spent and number of entries in central arena using Smart tracking software (San Diego instruments, San Diego, CA).

#### Elevated plus maze

The elevated plus maze (EPM) test was carried out according to the Walf and Frye protocol [19]. The EPM apparatus made of laminated wood and consisted of 2 opposing open arms (10×50 cm) and 2 opposing closed arms (10×50 cm with 30 cm high walls). The maze was placed 60 cm above the floor. White curtains surrounded the maze and behavior was recorded by an overhead video camera. Each rat was placed in the middle of the maze facing the open arm that faced away from the experimenter. The video camera recorded the time each rat spent in each of the arms over a period of 5 min. A closed arm entry was counted when the rat placed all four paws in a closed arm. An open arm entry was recorded when the rat placed all four paws in an open arm or when the rat's hind-limbs were placed in the central area of the maze and both fore-limbs in an open arm with its head protruding into the open arm.

### Protein analyses

Frontal cortex, hypothalamic and hippocampal tissues were homogenized in a lysis buffer using published protocol [14]. Levels of brain-derived neurotrophic factor (BDNF), Glucocorticoid receptor (GR), Neuropeptide Y-1 receptor (NPY-1R), Phopho-tropomyosin related kinase receptor type B (pTrkB), phopho-cyclic AMP-response element binding protein (pCREB), insulin receptor (IR-), phospho-insulin receptor substrate 1 (pIRS-1) were analyzed by Western blot. Protein samples were separated by electrophoresis on a 10% (12.5% for BDNF) polyacrylamide gel and electrotransferred to a PVDF or nitrocellulose membrane (Millipore, Bedford, MA). Non-specific binding sites were blocked with 5% non-fat dry milk in Tris-buffered saline (TBS) buffer containing 0.05% Tween-20 or 2% BSA in TBST. Membranes were rinsed in buffer (0.05% Tween-20 in TBS) and then incubated with anti-actin or anti-BDNF, anti-GR, anti-pTrkB, anti-TrkB, anti-IR-β, anti-pIRS-1 (Tyr 989), IRS-1 (1∶500; Santa Cruz Biotechnology, Santa Cruz, CA, USA), anti-pCREB(Ser133) and anti-CREB (1∶1000; Millipore, Bedford, MA), anti-NPY-1R (1∶500; Alpha Diagnostics International Inc. San Antonio, Texas) followed by anti-rabbit or anti goat or anti-mouse IgG horseradish peroxidase-conjugate (1∶10,000; Santa Cruz Biotechnology). The immunocomplexes were visualized by chemiluminescence using the ECL plus kit (Amersham Pharmacia Biotech Inc., Piscataway NJ, USA) for GR, NPY1R, pTrkB, pCREB, IR-β, pIRS-1, IRS-1 and SuperSignal West Femto kit (Thermo Scientific, Rockford, IL) for BDNF. Respective protein sizes were compared by using Bench mark pre-stained protein ladder (Invitogen Technology, Carlsbad, CA). The film signals were digitally scanned and then quantified using ImageJ software. Actin was used as an internal control.

### Fatty acid analysis by gas chromatography

Fatty acid profiles were determined by gas chromatography in whole cerebral cortical tissue-extracted lipids. The system consisted of model 5890A gas chromatograph (Hewlett Packard) and a model 7673A automatic, sampler and controller (Hewlett Packard). An Omegawax 250 column (30 m, 0.25-mm internal diameter, 0.25µm film thickness; Sigma-aldrich) was used, with helium as the carrier gas. GC oven temperature was initially held at 50°C for 2 min and raised with a gradient of 2°C min-1until 220°C and held for 30 min. The injector and detector were maintained at 250°C and 260°C, respectively. Tissues were reduced to powder under liquid nitrogen and subjected to extraction of total lipids. Fatty acid methylation was achieved by heating at 100°C for 1 hr with 14% boron tri-fluoride–methanol reagent. A 1 µ l sample of Fatty acid methyl esters (FAME) was injected in split injection mode with a 100∶1 split ratio. Peaks of resolved fatty acid methyl esters were identified and quantified by comparison with standards (Supelco 37-component FAME Mix). Identification of tissue fatty acids was carried out by comparison with the retention times of a standard mixture of fatty acid (682; MU-Chek-Prep, Elysian, MN). An internal standard (C23∶0 Me, tricosanoic acid methylester, 50–250 ug) was added to the sample (depending on the tissue size) before lipid extraction for the concentration of each fatty acid determination.

### Statistical analysis

Data were analyzed using statistics software (graph pad 5) and presented as means with their standard errors (SEM). Unpaired two-tailed t test was applied to compare the n-3 diet (n = 7) and n-3 def (n = 5) groups, in terms of protein and behavioral data. Protein results are expressed as mean percent of n-3 diet. The correlations between DHA levels and protein levels were analyzed by linear regression analysis. Criterion for significance was set to p<0.05 in all comparisons.

## Supporting Information

Table S1 **Composition of experimental diets.**
(DOC)
