# Supplementary material for: Omega-3 Fatty Acid Deficiency during Brain Maturation Reduces Neuronal and Behavioral Plasticity in Adulthood
Source: PLoS One. 2011 Dec 7;6(12):e28451. doi: 10.1371/journal.pone.0028451 (PMC3233581; doi:10.1371/journal.pone.0028451)
Supplement: Table S1 — Composition of experimental diets. (DOC) [file pone.0028451.s001.doc]

**Table S1:**

**Ingredient Amount (g/100 g diet)**

Alacid 710, acid casein 20 20

Cornstarch 15 15

Sucrose 10 10

Dextrose 19 19.9

Maltose-dextrin 15 15

Cellulose 5 5

Salt-mineral mix 3.5 3.5

Vitamin mix 1 1

L-cystine 0.3 0.3

Choline bitartrate 0.25 0.25

TBHQ 0.002 0.002

Fat sources: n-3 adq n-3 def

Hydrogenated coconut oil 7.45 8.1

Safflower oil 1.77 1.9

Flaxseed oil 0.48 none

DHA 1.2 none
